# Goose Astrovirus Type 2 Causes Intestinal Injury and Disrupts Homeostasis in Goslings

**DOI:** 10.3390/vetsci13010015

**Published:** 2025-12-23

**Authors:** Xinyang Li, Wenhao Yang, Ming Zhu, Haoran Xu, Junjie Yang, Zewen Yi, Yingjun Lv

**Affiliations:** College of Veterinary Medicine, Nanjing Agricultural University, Nanjing 210095, China; 2022807185@stu.njau.edu.cn (X.L.); yangwenhao@stu.njau.edu.cn (W.Y.); 2021107031@stu.njau.edu.cn (M.Z.); 2022107029@stu.njau.edu.cn (H.X.); 2025207013@stu.njau.edu.cn (J.Y.); 2020807153@stu.njau.edu.cn (Z.Y.)

**Keywords:** goose astrovirus type 2, intestinal damage, crypt cells, stem cells, signaling pathways

## Abstract

Goose astrovirus 2 (GAstV-2) infection leads to visceral gout and swollen kidneys in goslings, causing a 5–50% mortality rate and significant economic losses for goose flocks. While most studies on the virus’s pathological damage have focused on the kidneys, few reports have examined the effects of this fecal-oral pathogen on the digestive system. This study investigated GAstV-2 localization, cellular targets, and its impact on intestinal structure and homeostasis in orally infected goslings. Twenty 1-day-old goslings were randomly assigned to the infected and control groups. Clinical signs, organ lesions, viral distribution, histopathology, and alterations in intestinal cell populations, cytokine expression, and signaling pathways were assessed at 7 days post-infection. GAstV-2 was detected in the duodenum, jejunum, ileum, cecum, and rectum, with the highest viral load in duodenal crypt cells. Infection induced crypt cell necrosis, reduced villus height, decreased villus-to-crypt ratio, and lowered numbers of goblet cells and Lgr5+ intestinal stem cells. In contrast, Paneth cell abundance, Bmi1+ stem cells, and tight junction-related gene expression increased. Inhibition of stem cell differentiation into goblet cells was observed, mediated by modulation of the Notch signaling pathway. Proinflammatory cytokines, including *IL-1β*, *IL-6*, *IL-8*, *IL-22*, and *TNF-α*, were markedly upregulated, indicating a strong inflammatory response. These results demonstrate that GAstV-2 preferentially targets duodenal crypt cells, disrupts epithelial renewal, and impairs mucosal barrier function, while triggering compensatory regenerative and immune mechanisms. This study provides new insights into the intestinal pathogenesis of GAstV-2 and identifies potential targets for interventions to mitigate intestinal injury and economic losses in gosling production.

## Simple Summary

Goose astrovirus type 2 (GAstV-2) was first identified in Eastern China in 2018. This virus causes mortality rates of up to 50% in goslings aged 3 to 20 days, resulting in significant economic losses to the goose industry. Infected goslings primarily exhibit urate deposition on the surfaces of visceral organs and in joint cavities, pale and swollen kidneys, and lymphocyte depletion in the spleen. However, as a fecal-oral transmitted pathogen, the interactions between GAstV-2 and digestive system organs, as well as the associated pathological damage, remain poorly understood. In this study, we found that following GAstV-2 infection in goslings, the virus was detectable in all segments of the intestine but not in the esophagus, glandular stomach, or muscular stomach. The highest viral load was observed in the duodenum, where it induced crypt necrosis, shortened villus height, a reduced number of Lgr5+ stem cells, and increased expression of inflammatory cytokines. Conversely, the goslings responded to viral invasion by increasing the number of Paneth cells, enhancing the population of Bim+ stem cells, and upregulating the expression of tight junction proteins. These findings contribute to a better understanding of the pathogenic mechanisms of GAstV-2.

## 1. Introduction

Goose astrovirus (GAstV) is a non-enveloped, positive-sense, single-stranded RNA virus with a diameter of about 28–30 nm. Its genome, approximately 7.2 kb in length, consists of a 5′ untranslated region (UTR), three open reading frames (*ORF1a*, *ORF1b*, and *ORF2*), a 3′ UTR, and a poly(A) tail. *ORF1a* and *ORF2b* encode nonstructural proteins involved in RNA transcription and replication, such as RNA-dependent RNA polymerase (RdRP) and nuclear localization signal (NLS), while *ORF2* encodes capsid proteins associated with host immune responses [1,2]. Based on amino acid sequence differences in *ORF2*, GAstV is classified into two genotypes, GAstV-1 and GAstV-2, which share only 55.6–57.9% genome-wide similarity and 40.4–41.6% similarity in *ORF2* [3]. GAstV-2 has been identified as the primary causative agent of gout outbreaks in goslings, although GAstV-1 may also contribute, either alone or in co-infection with GAstV-2 [4,5,6]. First reported in 2018, GAstV-2 predominantly infects goslings aged 3–20 days, causing mortality rates of 5–50% in field cases and up to 93.1% in experimental infections [7,8,9,10]. Currently, there are no commercial vaccines or antiviral drugs available, leading to significant economic losses in goose production.

Gross lesions in affected goslings typically include swollen kidneys and urate deposition in the viscera and joints. Histopathological findings include renal tubular epithelial cell necrosis, hepatocyte degeneration, splenic lymphocyte depletion, and inflammatory cell infiltration [11,12]. Although GAstV-2 is transmitted via the fecal-oral route, most studies have focused on kidney pathology, with limited attention to its effects on the digestive system. The virus must traverse the intestinal barrier before reaching systemic circulation, yet the primary site of invasion, target cell types, and pathological changes in the gastrointestinal tract remain poorly characterized. Limited reports indicate GAstV-2 can be detected in the glandular stomach and duodenum and may alter the gut microbiota, increasing proinflammatory bacteria and reducing beneficial short-chain fatty acid producers [13,14]. These observations suggest that GAstV-2 may cause metabolic disturbances and intestinal injury, similar to astroviruses in other species such as human astrovirus, turkey astrovirus, and chicken astrovirus, which can damage intestinal epithelial cells, increase epithelial permeability, and impair growth [15,16,17].

Considering these knowledge gaps, the present study aimed to determine the distribution of GAstV-2 in the digestive tract of goslings, identify its target cell types, and characterize associated pathological and functional changes in the duodenum, with particular focus on intestinal stem cells, goblet cells, Paneth cells, inflammatory responses, tight junction integrity, and the involvement of Notch and Wnt signaling pathways.

## 2. Materials and Methods

### 2.1. Virus

The strain GAstV-2 (JSHA strain, GenBank: MK125058) used in this study was isolated and maintained in our laboratory. The virus titer, determined through titration on goose kidney epithelial cells using the Reed and Muench method, was 1 × 10^4.69^ TCID_50_/mL.

### 2.2. Animal Experiments

1-day-old Yangzhou white goose goslings, supplied by Nanjing Sanhua Goose Company Ltd. (Nanjing, China), were used in this study. Prior to the experiment, five goslings were euthanized via intravenous administration of pentobarbital sodium for baseline health assessment. Necropsy revealed no gross pathological lesions in all examined organs, and GAstV-2 RNA was not detected by RT-PCR in the kidney, spleen, or liver tissues. Subsequently, twenty 1-day-old goslings were randomly allocated into two groups: an infected group and a negative control group (n = 10 per group). All subjects were maintained in isolated negative-pressure housing under standardized conditions. They were supplied with sterilized water and a nutritionally identical, antibiotic-free diet ad libitum. The infected group received an oral challenge comprising 0.8 mL of GAstV-2 viral suspension (0.8 × 10^4.69^ TCID_50_/goose), whereas control animals were administered an equivalent volume of PBS via the identical route. Clinical signs were recorded daily after infection. Any mortalities were subjected to immediate necropsy, and gross lesions were documented. At 7 days post-infection (dpi), all surviving goslings were euthanized by intravenous injection of pentobarbital sodium following body weight measurement. The kidneys, livers, and spleens were weighed to determine relative organ weights (organ-to-body weight ratio). A portion of the kidneys, esophaguses, glandular stomachs, muscular stomachs, duodenums, jejunums, ileums, ceca, and rectums was fixed in 4% paraformaldehyde for histopathological evaluation, while the remainder was preserved at −80 °C for further analysis. Each gosling was considered to be a replicate in this study.

### 2.3. Histopathological Analysis

Histopathological examination was performed as previously described [11]. Briefly, fixed tissue samples were dehydrated through a graded alcohol series, cleared in xylene, and embedded in paraffin. Serial sections (4 μm thick) were then prepared and stained for examination under a light microscope (Carl Zeiss, Munich, Germany).

### 2.4. Periodic Acid-Schiff (PAS) and Phloxine B Paneth Cell Staining

Duodenal sections were stained with PAS and Phloxine B for Paneth cell staining to detect goblet and Paneth cells using commercial kits (Beyotime, Shanghai, China). Twenty-five random, non-overlapping high-powered fields of duodenum tissue at 400-fold magnification were chosen to count the number of PAS-positive cells. Paneth cells exhibited pink staining, and the positive signals were semi-quantitatively analyzed using ImageJ software (version 1.54g).

### 2.5. Virus Determination

Viral RNA was extracted from kidney and intestine samples using the RNeasy Isolation Kit (Vazyme, Nanjing, China). Subsequently, RNA was reverse-transcribed into cDNA with the HiScript Q RT SuperMix Kit (Vazyme, Nanjing, China) in accordance with the manufacturer’s instructions. Viral loads were then quantified via a SYBR Green I-based real-time PCR following a previously established protocol from our laboratory [18].

### 2.6. Quantitative Real-Time PCR (qRT-PCR) Analysis

Total RNA was extracted from duodenal tissue samples using RNA-easy Isolation Reagent. According to the manufacturer’s protocol, the extracted RNA was reverse-transcribed into cDNA using the HiScript Q RT SuperMix Kit. Quantitative real-time PCR (qPCR) was conducted on an AB7300 thermal cycler (Life Technologies, Carlsbad, CA, USA). Specific primers for the following target genes were designed with Primer 5.0 software: *Lyz*, *Muc2*, *Lgr5*, *Bmi1*, *OLFM4*, *Wnt3A*, *Axin2*, *AhR*, *cycD*, *Myc*, *Jun*, *Notch1*, *Hes1*, *Dll4*, *Jag1*, *Hey1*, *NRARP*, *ATOH1*, *ZO-1*, *ZO-2*, *Claudin-1*, *Claudin-2*, *IL-1β*, *IL-6*, *IL-8*, *IL-22*, *TNF-α,* and *iNOS*. Gene expression levels were quantified using the 2^−ΔΔCT^ method and normalized to GAPDH as the endogenous control. All reactions were performed in triplicate. Primer sequences are provided in Supplementary Table S1.

### 2.7. Immunohistochemical (IHC) Examination

IHC staining was performed following a previously described protocol with slight modifications [19]. Briefly, tissue sections were deparaffinized, rehydrated, and treated with 3% H_2_O_2_ for 10 min to inactivate endogenous peroxidase. Antigen retrieval was performed by heating the sections in citrate buffer at 100 °C for 8 min. Non-specific binding was blocked with 5% bovine serum albumin for 30 min. Sections were then incubated overnight at 4 °C with rabbit anti-GAstV-2 Capsid protein polyclonal antibody and *Lgr5* Rabbit polyclonal antibody (Beyotime, Shanghai, China) as the primary antibody. After washing with PBS, sections were incubated with secondary antibodies for 1 h at 37 °C. Staining was visualized using 3,3′-diaminobenzidine (DAB) substrate, followed by counterstaining with hematoxylin for 8 min. Stained sections were examined under a light microscope, and positive signals were semi-quantitatively analyzed using ImageJ software (version 1.54g). Briefly, for each tissue section, 25 non-overlapping fields of view containing positive signals were randomly selected at 400× magnification. Within each field, the area of positive signals (brown deposition) was measured, and the percentage of positive area relative to the total field area was calculated. The values obtained from the 25 fields per sample were averaged to determine the mean optical density (MOD), which served as a quantitative indicator of target protein expression.

### 2.8. Statistical Analysis

The differences between the control group and experimental group were analyzed by Student’s *t*-test using SPSS software 16.0. Data are presented as mean ± standard deviation (SD). Statistical significance was defined as *p* < 0.05.

## 3. Results

### 3.1. Clinical Changes and Confirmation of GAstV-2 Infection

**Figure 1:**
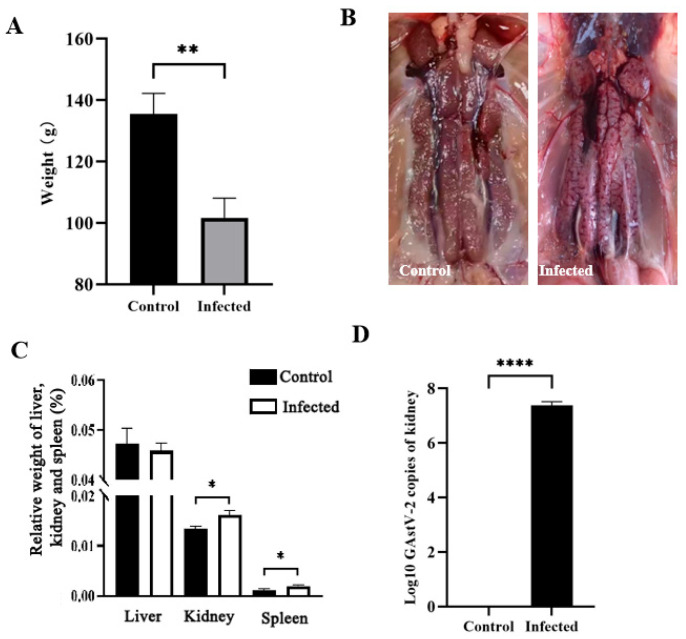
Changes in body weight (**A**), kidney lesions (**B**), relative weights of kidney, liver, and spleen (**C**), and viral load in the kidney (**D**) of goslings infected with GAstV-2 at 7 dpi. Values are expressed as mean ± SD (control group: n = 10; infected group: n = 9). * *p* < 0.05; ** *p* < 0.01; **** *p* < 0.0001.

The goslings in the infected group exhibited depression, reduced feed intake, and mild diarrhea, with a mortality rate of 10%. No clinical signs were observed in the control group. At 7 days post-infection (dpi), body weight in the infected group was significantly lower than that in the control group (*p* < 0.05; Figure 1A). Gross examination revealed swollen kidneys and urate deposition in infected goslings, whereas no lesions were present in controls (Figure 1B). Relative kidney and spleen weights were significantly higher in infected birds than in controls (*p* < 0.05; Figure 1C). Viral loads in the kidneys of infected goslings reached 10^7^ copies/μL, whereas no viral RNA was detected in controls (Figure 1D), confirming successful GAstV-2 infection.

### 3.2. Distribution and Localization of GAstV-2 in Digestive Tissues

**Figure 2:**
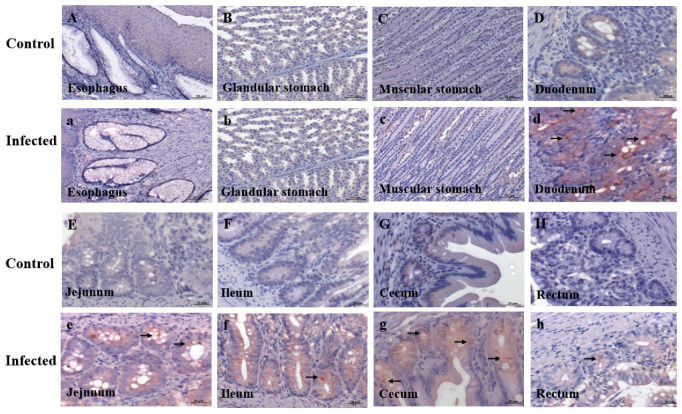
The virus location was detected by IHC in different digestive tissues of goslings infected with GAstV-2 at 7 dpi. Esophagus, glandular stomach, muscular stomach, duodenum, jejunum, ileum, cecum, and rectum were stained in the control (**A**–**H**) and infected goslings (**a**–**h**). Brown positive signals are marked with black arrows.

**Figure 3:**
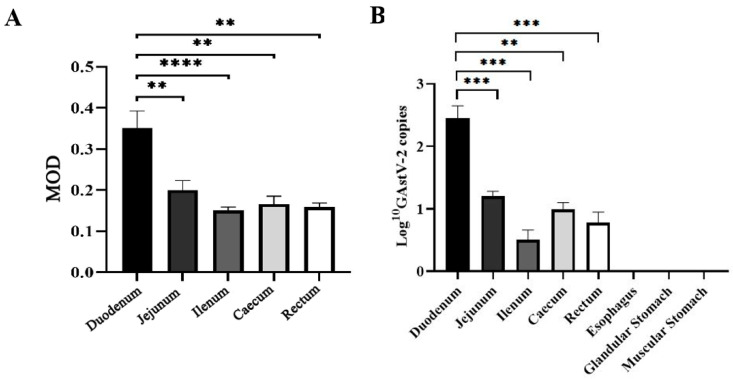
Viral load in duodenum, jejunum, ileum, cecum, and rectum detected by IHC (**A**) and qRT-PCR (**B**). Values are expressed as mean ± SD (control group: n = 10; infected group: n = 9). ** *p* < 0.01; *** *p* < 0.001; **** *p* < 0.0001.

**Figure 4:**
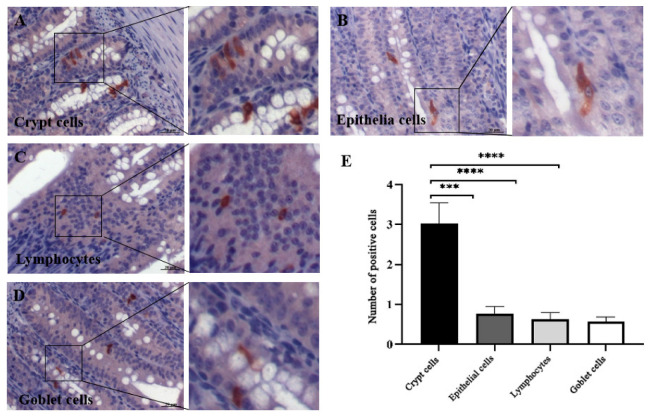
Virus localization and quantification of GAstV-2 in different cell types of the duodenum. The virus was localized in crypt cells (**A**), intestinal epithelial cells (**B**), lymphocytes (**C**) of the lamina propria, and goblet cells (**D**). The positive cell number per high-power field is shown (**E**). Values are expressed as mean ± SD (control group: n = 10; infected group: n = 9). *** *p* < 0.001; **** *p* < 0.0001.

Immunohistochemistry (IHC) detected GAstV-2 antigens (brown deposition) in the duodenum, jejunum, ileum, cecum, and rectum of infected goslings, with no signals in the esophagus, glandular stomach, or muscular stomach. No positive signals were observed in any digestive tissue of controls (Figure 2). Quantitative analysis showed the strongest IHC signal intensity in the duodenum among all intestinal segments (*p* < 0.01) (Figure 3A). Consistent with these findings, qRT-PCR revealed that the viral load was highest in the duodenum compared to all other intestinal segments (*p* < 0.01), while no detectable virus was found in the esophagus, glandular stomach, or muscular stomach (Figure 3B). Within the duodenum, viral antigens were localized to crypt cells, intestinal epithelial cells, lamina propria lymphocytes, and goblet cells, with crypt cells harboring the greatest viral load (*p* < 0.01) (Figure 4).

### 3.3. Histopathological Changes in the Duodenum

**Figure 5:**
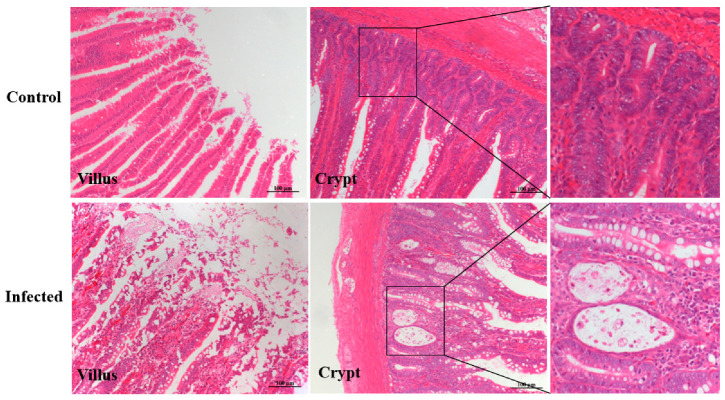
Histopathological changes in the duodenum of goslings infected with GAstV-2 at 7 dpi.

Hematoxylin and eosin staining revealed crypt cell necrosis characterized by karyopyknosis and karyorrhexis, along with mild villus shedding in infected goslings, whereas no histological abnormalities were observed in controls (Figure 5).

### 3.4. Effect of GAstV-2 on Villus Height, Crypt Depth, Goblet Cells, and Mucus Production

**Figure 6:**
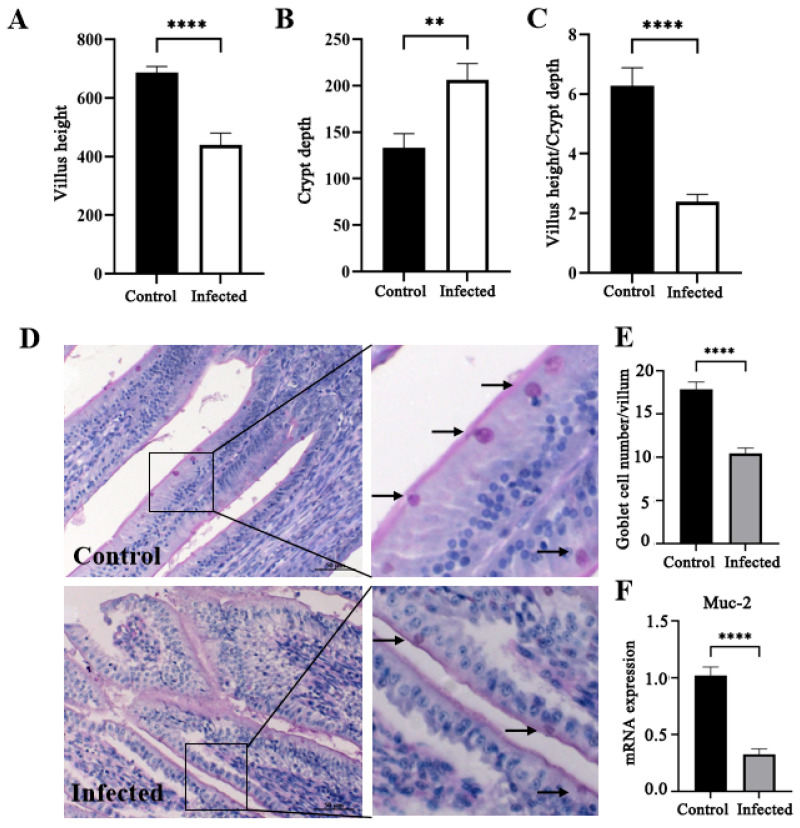
Changes in villus height (**A**), crypt depth (**B**), villus height-to-crypt depth ratio (**C**), PAS-stained goblet cells (indicated by black arrow) (**D**), goblet cell number (**E**), and Muc-2 mRNA expression (**F**) in the duodenum of goslings infected with GAstV-2 at 7 dpi. (**D**). Values are expressed as mean ± SD (control group: n = 10; infected group: n = 9). ** *p* < 0.01; **** *p* < 0.0001.

The infected goslings exhibited significantly reduced villus height, increased crypt depth, and a decreased villus height-to-crypt depth ratio compared with the controls (*p* < 0.05; Figure 6A–C). Goblet cell counts and *Muc-2* mRNA expression were markedly lower in the infected birds (*p* < 0.05; Figure 6D–F).

### 3.5. Effect of GAstV-2 on Paneth Cells and Intestinal Stem Cells

**Figure 7:**
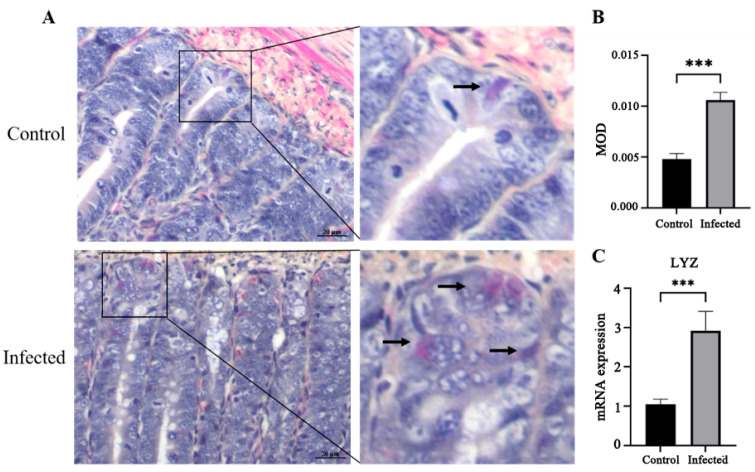
Effect of GAstV-2 infection on Paneth cells in the duodenum. Paneth cells were stained with Phloxine B and are indicated by black arrows (**A**), and the mean optical density (MOD) was calculated using ImageJ software (version 1.54g) (**B**). Lyz mRNA expression was detected by qRT-PCR (**C**). Values are expressed as mean ± SD (control group: n = 10; infected group: n = 9). *** *p <* 0.001.

**Figure 8:**
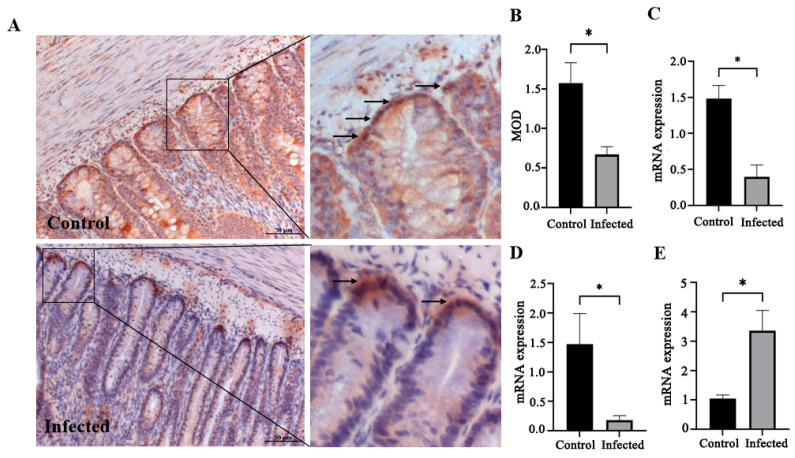
Effect of GAstV-2 infection on Lgr5+ and Bmi 1+ cells in the duodenum. Lgr5+ cells were detected by IHC and are indicated by black arrows (**A**), and MOD was calculated using ImageJ software (version 1.54g) (**B**). The mRNA expressions of *Lgr5* (**C**), *OLFM4* (**D**), and *Bmi1* (**E**) were measured by qRT-PCR. Values are expressed as mean ± SD (control group: n = 10; infected group: n = 9), * *p* < 0.05.

Phloxine B staining and quantitative analysis showed a significant increase in Paneth cell abundance in infected goslings compared with controls (*p* < 0.05; Figure 7A,B). Similarly, *Lyz* mRNA expression was elevated (*p* < 0.05; Figure 7C). IHC revealed that Lgr5+ ISCs were significantly reduced in number in infected goslings (*p* < 0.05; Figure 8A,B), accompanied by decreased mRNA expression of *Lgr5* and *OLFM4* (*p* < 0.05; Figure 8C,D). Conversely, *Bmi1* mRNA expression was significantly increased in infected goslings (*p* < 0.05; Figure 8E).

### 3.6. Effect of GAstV-2 on Notch and Wnt Signaling Pathways

**Figure 9:**
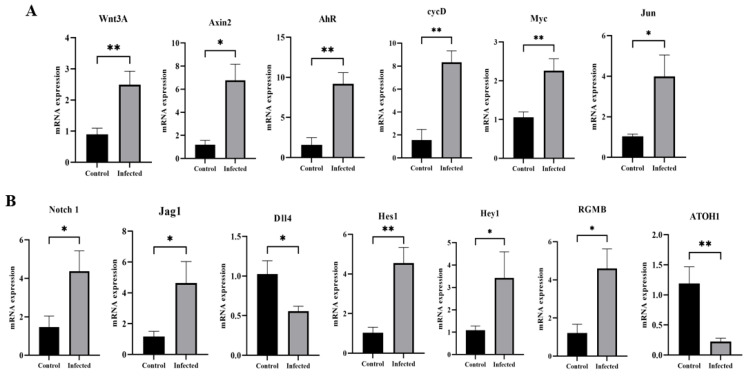
Changes in mRNA expression of Wnt (**A**) and Notch (**B**) signaling pathway-related genes in the duodenum of goslings infected with GAstV-2 at 7 dpi. Values are expressed as mean ± SD (control group: n = 10; infected group: n = 9). * *p* < 0.05; ** *p* < 0.01.

qRT-PCR analysis indicated that GAstV-2 infection significantly upregulated the mRNA expression of Wnt pathway genes *Wnt3A*, *Axin2*, *cycD*, *AhR*, *Myc*, and *Jun* (*p* < 0.05; Figure 9A). Notch pathway-related genes, including *Notch1*, *Jag1*, *Hes1*, *Hey1*, and *NRARP*, were also significantly upregulated in infected goslings, while *Dll4* and *ATOH1* were downregulated (*p* < 0.05; Figure 9B).

### 3.7. Effect of GAstV-2 on Inflammatory Cytokines and Tight Junction-Associated Genes

**Figure 10:**
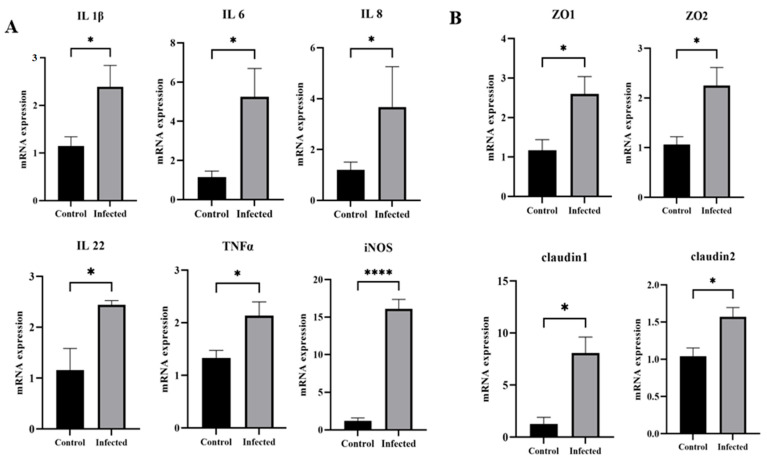
Changes in mRNA expression of cytokine genes (**A**) and tight junction–related genes (**B**) in the duodenum of goslings infected with GAstV-2 at 7 dpi. Values are expressed as mean ± SD (control group: n = 10; infected group: n = 9). * *p* < 0.05; **** *p* < 0.0001.

Infected goslings displayed significant increases in the mRNA expression of proinflammatory cytokines *IL-1β*, *IL-6*, *IL-8*, *IL-22*, *TNF-α*, and *iNOS* (*p* < 0.05; Figure 10A). Expression of tight junction-related genes *ZO-1*, *ZO-2*, *Claudin-1*, and *Claudin-2* was also significantly elevated compared with controls (*p* < 0.05; Figure 10B), indicating that GAstV2 infection caused inflammation and enhanced tight junction expression in the duodenum.

## 4. Discussion

GAstV-2 is transmitted via the fecal–oral route, yet its localization and pathogenic effects within the digestive system are not well defined. In this study, oral inoculation of goslings with GAstV-2 resulted in reduced body weight, pale and swollen kidneys, and urate deposition, confirming the successful establishment of a gout model in goslings. Viral antigens were detected in the duodenum, jejunum, ileum, and cecum, but not in the esophagus, glandular stomach, or muscular stomach. These results indicate a clear intestinal tropism, suggesting the intestine may be the primary site of invasion following oral exposure. The duodenum harbored the highest viral load, which may be attributable to its C-shaped structure that favors viral retention or to its strong digestive and absorptive capacity. In contrast, Wei et al. reported GAstV-2 localization in the glandular stomach, a difference that may reflect variation in viral strain or experimental conditions [13]. It is also possible that after a single oral challenge, GAstV-2 transits rapidly through the glandular stomach to the intestine, limiting gastric epithelial contact. Additional studies are needed to determine whether the glandular stomach can serve as a site of GAstV-2 replication.

Our experiments further revealed that GAstV-2 localizes to intestinal crypt cells, goblet cells, intestinal epithelial cells, and lymphocytes, with the highest viral content detected in crypt cells. This distribution is consistent with infection patterns reported for astroviruses in other species: HAstV can infect intestinal epithelial cells [15], murine astrovirus localizes to goblet cells [20], and CAstV targets crypt epithelial cells [17]. Our previous work also detected GAstV-2 antigens in the cytoplasm of peripheral blood lymphocytes [21]. Furthermore, CAstV has been reported to replicate within crypts [17]. To date, there have been no reports describing GAstV-2 invasion of intestinal target cells, and it remains to be determined whether GAstV-2 can replicate within crypts.

Crypt cells play a vital role in maintaining intestinal barrier function. Located at the base of the crypt are ISCs and Paneth cells. ISCs are essential for sustaining intestinal epithelial homeostasis through continuous self-renewal and support of the rapid epithelial turnover. ISCs can be classified into two distinct populations: active ISCs, which are marked by the expression of *Lgr5*, and quiescent ISCs, identified by the expression of Bmi1. Lgr5+ ISCs divide regularly under steady-state conditions and are highly sensitive to injury or stress, whereas Bmi1+ ISCs contribute little to epithelial renewal during homeostasis but are activated to mediate regeneration following damage [22]. Paneth cells perform multiple functions, including antibacterial defense through the secretion of antimicrobial proteins such as lysozyme, regulation of intestinal homeostasis, and support of ISC maintenance. In this study, GAstV-2 infection resulted in a reduction in Lgr5+ ISCs, accompanied by increases in both Bmi1+ ISCs and Paneth cells. These findings suggest that GAstV-2 damages Lgr5+ ISCs, and that goslings respond by enhancing resistance to infection and promoting epithelial regeneration through increased numbers of Paneth cells and Bmi1+ ISCs.

ISCs generate transit-amplifying (TA) progenitors, which undergo terminal differentiation into specialized intestinal cell types such as enterocytes, goblet cells, and Paneth cells under the regulation of the Notch signaling pathway. Inhibition of Notch signaling has been shown to promote the development of secretory progenitor cells by upregulating the atonal basic helix-loop-helix (bHLH) transcription factor 1 (*ATOH1*) [23,24]. In this study, GAstV-2 infection activated the Notch signaling pathway while significantly reducing *ATOH1* mRNA expression, indicating that GAstV-2 suppresses ISC differentiation into goblet cells. This finding is supported by the observed histopathological decrease in goblet cell numbers and the reduced expression of the *Muc-2* gene. Wnt signaling is another key regulatory pathway for ISCs and plays a decisive role in determining their fate during epithelial repair following injury [25]. Here, GAstV-2 infection also activated the Wnt signaling pathway, which may contribute to the regeneration of damaged epithelial cells.

Tight junctions play a critical role in host defense as part of the innate immune barrier. In this study, GAstV-2 infection upregulated the expression of *ZO* and *Claudin* genes, suggesting that tight junctions may help limit viral invasion during infection. In contrast, HAstV-1 and TAstV-2 have been shown to disrupt tight junction complexes, increasing intestinal permeability and leading to diarrhea [26,27]. These findings suggest that different astrovirus species may exert distinct regulatory effects on intestinal tight junctions, potentially reflecting host-specific or virus-specific adaptations. Indeed, phylogenetic analysis of complete genomic sequences placed GAstV-2 within a distinct clade, separate from astroviruses infecting humans, turkeys, and chickens. Comparative sequence alignments further revealed low genetic similarity, with nucleotide and amino acid identity below 65% relative to these viruses [15,28,29]. Moreover, GAstV-2 exhibits notable differences in pathogenicity. While turkey and human astroviruses primarily cause intestinal damage and diarrhea, GAstV-2 infection typically results in kidney injury in geese. The intestine may serve primarily as the entry site for GAstV-2, and the upregulation of tight junction-related genes likely represents a host defense response against viral invasion.

Cytokine production is a key component of the host immune response to viral infection and plays an important role in immune regulation. However, excessive cytokine release can lead to tissue damage and aggravation of clinical symptoms. In this study, GAstV-2 infection triggered robust production of proinflammatory cytokines, which may contribute to intestinal injury. Similar cytokine induction by GAstV-2 has been reported in the blood, kidneys, and spleen [14,30,31], indicating that this response is part of a broader systemic inflammatory reaction during infection.

## 5. Conclusions

In conclusion, following oral infection of goslings with GAstV-2, the virus initially targets the duodenum, localizing predominantly in intestinal crypts. This leads to crypt cell necrosis, reduced villus height, and decreased numbers of Lgr5+ ISCs, along with inhibition of ISC differentiation into goblet cells through modulation of the Notch signaling pathway. These changes, coupled with an enhanced inflammatory response, contribute to intestinal injury. Conversely, infected goslings exhibit increased numbers of Paneth cells and Bmi1+ ISCs, upregulation of tight junction–associated genes, and activation of the Wnt signaling pathway, which may enhance resistance to infection and promote epithelial regeneration. Collectively, these findings provide new insight into GAstV-2–host interactions in the intestine and advance our understanding of the pathogenic mechanisms underlying GAstV-2 infection.

## Data Availability

The original contributions presented in this study are included in the article. Further inquiries can be directed to the corresponding author.

## Supplementary Materials

The following supporting information can be downloaded at: https://www.mdpi.com/article/10.3390/vetsci13010015/s1. Table S1 Primers used in this study.
