# Supplementary material for: Goose Astrovirus Type 2 Causes Intestinal Injury and Disrupts Homeostasis in Goslings
Source: Vet Sci. 2025 Dec 23;13(1):15. doi: 10.3390/vetsci13010015 (PMC12846611; doi:10.3390/vetsci13010015)
Supplement: Supplementary file 1 [file vetsci-13-00015-s001.zip › vetsci-4060138-supplementary.pdf]

Table S1 Primers used in this study

Product	Genbank ID	Prime sequences(5′→3′)	Product size
GAPDH-F	MG674174.1	TCAAGGCTGAGAATGGGAAAC	191 bp
GAPDH-R		GGCGGAGATGATGACACG	
Lgr5-F	XM_013197953.1	AAAGATGTTGGGTTGTTAC	121 bp
Lgr5-R		TGAAGGGACCTGGAGAAG	
Bmi1-F	XM_013171771.1	GGAGAAGTGGCTGACGAA	159 bp
Bmi1-R		TGGGCAGCGTAAGTATCT	
OLFM4-F	XM_013179556.2	CCTGCATTCACGGTGGACTA	124 bp
OLFM4-R		TCCTTCTCTGGGTCAGAGGG	
Lyz-F	XM_013198195.1	CTATGAAGCGACTTGGACTG	102 bp
Lyz-R		GGTTTGTAGCCTGCGTGT	
Muc2-F	XM_038180256.1	CACCTCCTAAACCCACAT	161 bp
Muc2-R		GCAATTCCCTTGATAGCT	
Wnt3A-F	XM_013182510.1	TGCTTCTGAAATGGTGGTG	164 bp
Wnt3A-R		TCCCGAAGGAACCTGTCT	
Axin2-F	XM_013178833.1	GCCCAGAGCCTGACACTT	112 bp
Axin2-R		CTTCAAACACTGCACCACA	
AhR-F	XM_013171792.1	TCCTTACAAGCAACCCAC	169 bp
AhR-R		AGAGTTTATCCCACAATCA	
cycD-F	XM_048070817.1	CTTGGATGCTGGAGGTTTGC	109 bp
cycD-R		CGGCTTTTCTTGAGGGGTTC	
Myc-F	XM_013184287.1	TCAAGTCCCCGCACGTCAGA	112 bp
Myc-R		GGTCACGCAACGCAAAGAAA	
Jun-F	XM_013176141.2	GAGAATCAAAGCCGAGCGAAAG	154 bp
Jun-R		TCTGAGCATGTTGGCAGTGGA	
Notch1-F	XM_013188670.1	GCGAATGGGATGGTCTGG	299 bp
Notch1-R		GAGGCTGGTCTTTACTTTGTTG	
Hes1-F	XM_027464446.2	CGCCCGATAAGCCCAAGAC	148 bp
Hes1-R		GAGTGCCGCGAGCTATCCTTC	
Dll4-F	XM_027459564.2	CTGGTGGCTTTGCTCATA	211 bp
Dll4-R		GATTCTTGGGTTTGTAGTTTG	
Jag1-F	XM_027453356.2	TTGTCGTCAGGGATGTAG	211 bp
Jag1-R		GTGGGTTCCGCAGTAGTT	
Hey1-F	XM_048068396.1	CGTCGACCACCTGAAAATGC	89 bp
Hey1-R		ACCCTAAGCTCCGGTAGTCC	
NRARP-F	XM_048056945.1	AAGCTGCTCGTCAAGTTCGG	116 bp
NRARP-R		ATTTGGCCTTGGTGATCAGGT	
ATOH1-F	XM_004941130.5	CGACCAGCTGCGTAATGTCA	72 bp
ATOH1-R		ATCTGCAGCGTCTCGTACTT	
ZO1-F	XM_013177403.1	TACGCTGTTGAATGTCCC	175 bp
ZO1-R		ATGGTCTGAAGGCTCTGA	

Continued Table S1 Primers used in this study

Product	Genbank ID	Prime sequences(5′→3′)	Product size
ZO2-F	XM_013194031.1	ACCACCACCTGTTTCTAT	186 bp
ZO2-R		ATCCTTGCCTTATGACCC	
claudin1-F	XM_048057903.1	TGATGGTGGCTGCGATAC	218 bp
claudin1-R		AACAGGCGTGAAAGGGTC	
claudin2-F	XM_048077173.1	CACCAAGTACGAGATGGGGG	131 bp
claudin2-R		GGTAGGCGTTCGAGTAGGTG	
IL1β-F	JF505290.1	TCCGCCAGCCGCAAAGTG	136 bp
IL1β-R		CGCTCATCACGCAGGACA	
IL6-F	XM_048070285.1	AAGCATCTGGCAACGACGAT	149 bp
IL6-R		TCTCGGAGGATGAGGTGTGT	
IL8-F	AB213393.1	CCTGATTTCCGTGGCTCTGT	135 bp
IL8-R		CCACTTTGCGTCAGCTTCAC	
IL22-F	XM_013196285.1	GTTCCTGGCATCCCTGAC	202 bp
IL22-R		GTTCCCTCCCTTCTTTGG	
TNFα-F	EU375296.1	GAATGAACCCTCCTCCG	139 bp
TNFα-R		ATCTGGTTACAGGAAGG	
iNOS-F	U34045.1	GAACAGCCAGCTCATCCGATA	103 bp
iNOS-R		CCCAAGCTCAATGCACAACTT	
